# Catalyst-Free Amino-Yne Click Reaction: An Efficient Way for Immobilizing Amoxicillin onto Polymeric Surfaces

**DOI:** 10.3390/polym16020246

**Published:** 2024-01-15

**Authors:** Julia Sánchez-Bodón, Maria Diaz-Galbarriatu, Rebeca Sola-Llano, Leire Ruiz-Rubio, José Luis Vilas-Vilela, Isabel Moreno-Benitez

**Affiliations:** 1 Macromolecular Chemistry Group (LABQUIMAC), Department of Physical Chemistry, University of the Basque Country (UPV/EHU), 48940 Leioa, Spain; julia.sanchez@ehu.eus (J.S.-B.); mariadiazg@ehu.eus (M.D.-G.); leire.ruiz@ehu.eus (L.R.-R.); joseluis.vilas@ehu.eus (J.L.V.-V.); 2 Department of Physical Chemistry, University of the Basque Country (UPV/EHU), 48940 Leioa, Spain; rebeca.sola@ehu.eus; 3 BCMaterials, Basque Center for Materials, Applications and Nanostructures, UPV/EHU, Science Park, 48940 Leioa, Spain; 4 Macromolecular Chemistry Group (LABQUIMAC), Department of Organic and Inorganic Chemistry, University of the Basque Country (UPV/EHU), 48940 Leioa, Spain

**Keywords:** click chemistry, amino-yne, bioconjugation, polymer

## Abstract

Surface modifications play a crucial role in enhancing the functionality of biomaterials. Different approaches can be followed in order to achieve the bioconjugation of drugs and biological compounds onto polymer surfaces. In this study, we focused on the immobilization of an amoxicillin antibiotic onto the surface of poly-L-lactic acid (PLLA) using a copper-free amino-yne click reaction. The utilization of this reaction allowed for a selective and efficient bioconjugation of the amoxicillin moiety onto the PLLA surface, avoiding copper-related concerns and ensuring biocompatibility. The process involved sequential steps that included surface activation via alkaline hydrolysis followed by an amidation reaction with ethylendiamine, functionalization with propiolic groups, and subsequent conjugation with amoxicillin via a click chemistry approach. Previous amoxicillin immobilization using tryptophan and fluorescent amino acid conjugation was carried out in order to determine the efficacy of the proposed methodology. Characterization techniques such as X-ray photoelectron spectroscopy (XPS), Attenuated Total Reflection (ATR)–Fourier Transform Infrared (FTIR) spectroscopy, surface imaging, water contact angle determination, and spectroscopic analysis confirmed the successful immobilization of both tryptophan and amoxicillin while maintaining the integrity of the PLLA surface. This tailored modification not only exhibited a novel method for surface functionalization but also opens avenues for developing antimicrobial biomaterials with improved drug-loading capacity.

## 1. Introduction

Polylactic acid (PLA), an aliphatic polyester derived from starch (2-hydroxypropanoic acid), stands as a highly versatile and biodegradable polymer with unique physicochemical properties, including processability and transparency [1,2,3]. The stereoisomer poly-L-lactic acid (PLLA) has garnered attention for exceptional biocompatibility and mechanical strength, which shows promise in large-tissue regeneration [4,5,6]. Moreover, the versatility in processing PLLA-based biomaterials using diverse techniques such as extrusion, injection molding, film casting, thermoforming, foaming, fiber spinning, electrospinning, and melt electrospinning (as well as micro- and nanofabrication methods) has been essential in broadening the scope of applications for these substrates [7]. For instance, PLLA-based materials find extensive use in orthopedics and dentistry for fixation devices such as screws, pins, or washers, among others. These biomaterials offer some advantages compared to non-biodegradable implants; in fact, they do not require additional removal surgeries, as they degrade in biological media [7]. However, their slow degradation and high crystallinity can potentially cause inflammatory reactions in the body [8]. Furthermore, their hydrophobicity can induce biofilm formation, as bacteria tend to adhere to flat and hydrophobic surfaces [9,10]. Indeed, the issue of microbial contamination concerns multiple sectors ranging from food packing to medical devices [11]. Despite sterilization efforts using heat or radiation [12], polymers remain susceptible to microbial colonization, leading to potential infections upon exposure. In fact, bacterial adhesion is the initial step in biomaterial-related infections and serves as a foundation for subsequent implant colonization. Once attached, pathogens form microcolonies and develop protective biofilms, allowing them to persist in hostile hot environments [13,14,15]. This vulnerability requires the development of antimicrobial materials to avoid microbial growth and prevent subsequent colonization and proliferation. Recent advancements have been described that include the integration of additives such as drugs, natural compounds, or peptides to further enhance the biological properties of these biomaterials [4].

Surface immobilization and functionalization represent pivotal methodologies for creating functional surfaces with a wide range of properties [16,17,18,19]. Several strategies are involved in surfaces functionalization that can be categorized as non-covalent and covalent based on the interaction type between the molecular species and surfaces. It has to be noted that previous activation of the surface (usually an oxide layer surface) is required for posterior functionalization. Developing a robust ligation reaction that offers high efficiency and a simple procedure is highly desirable. In fact, this type of chemical functionalization serves as a biofunctional platform for biosensing, tissue engineering, and other chemical and biological applications [20,21,22,23,24]. Many covalent modifications have been reported for surface immobilization that encompass ester-amino ligation through a carbodiimide procedure, aldehyde-assisted ligation, epoxy-amino ligation, thiol–maleimide reactions, a Staudinger reaction, or photoactive bioconjugations [21,25,26]. Nevertheless, most of them are easily hydrolysable or they require complicated procedures that are inefficient and that limit their applications in some media. However, the development of click reactions (in particular, a metal catalyst-free amino-yne click reaction) has become a useful tool in bioconjugation–immobilization approaches [27].

Click chemistry has revolutionized different science areas by facilitating the formation of C-C or C-heteroatom bonds with remarkable efficiency and selectivity. These reactions stand out due to their ability to produce high yields without requiring an extensive purification process of the obtained products [28,29]. This innovative methodology represents a versatile and valuable approach that seamlessly constructs complex structures by merging smaller molecules through a concise set of highly efficient reactions [30,31,32,33,34]. Hence, their use has rapidly garnered prominent attention in different research fields encompassing organic chemistry, polymer and materials science, optics, engineering, and biomedical research [30,35,36]. The 1,3-dipolar cycloaddition between azides and alkynes emerged as the prototype reaction among those classified as “click”. Moreover, the inertness of alkynes and azides toward various environmental factors and reactive species makes them suitable for applications in biological systems. While this reaction is widely recognized for its straightforward selectivity of these functional groups, its true value lies in the use of copper as catalyst, increasing the reaction rate up to 10^7^ [33,34,37,38]. Nevertheless, it is crucial to note that copper, while serving as a catalyst, can potentially affect cell viability by inducing cytotoxicity [39]. In recent years, several strategies have been developed to minimize the dependency on this catalyst. For instance, Bertozzi and collaborators introduced systems involving cyclooctynes in which the reaction between the cyclic alkyne and azide occurs in a physiological environment [40,41]. The focus of this chemistry lies in the highly strained, medium-sized cyclic alkyne and, in particular, cyclooctynes. This instability in cyclic alkynes is directly related to the C-C triple bond angle, which, due to the cyclic structure, cannot achieve the ideal 180° bond angle observed in sp-hybridized carbon atoms. Unlike linear acetylenes, the deformation of the carbon–carbon triple bond of a cyclooctyne allows the spontaneous reaction with an azide compound despite its poor ability to act as a 1,3-dipolar acceptor [40,42,43]. While azide–alkyne reactions offer orthogonality advantages, they do come with certain limitations. The azide functional group is relatively uncommon in drugs or biologically active compounds, often requiring prior compound derivatization. Additionally, while the strain-promoted cycloaddition of cyclooctynes with azides can serve as a versatile and copper-free alternative to the widely used CuAAC click reaction, the synthesis of these cyclooctynes involves challenging functionalization steps and an uneconomical strategy [43].

**Scheme 1:**
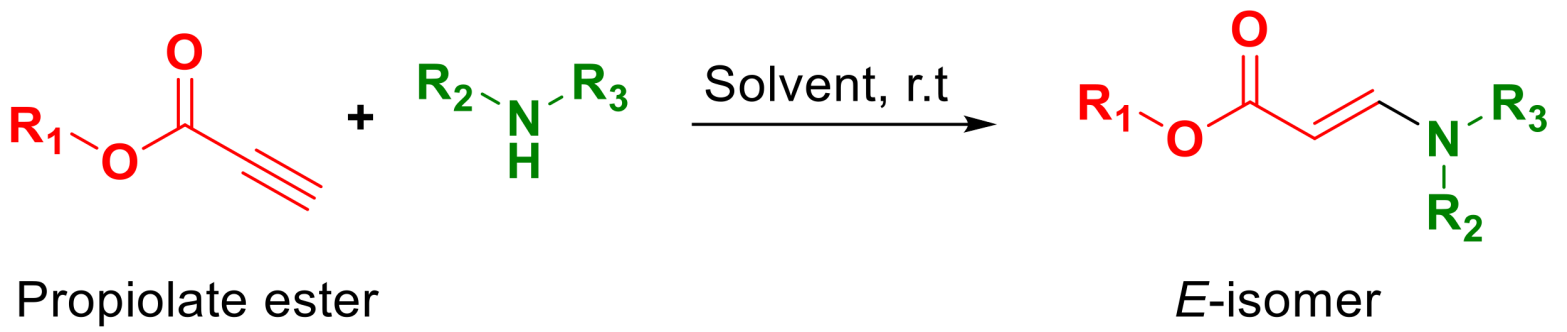
Illustrative scheme of spontaneous amino-yne click reaction.

Recent strategies have been reported that can overcome these issues. Among these innovative click methodologies [20], the spontaneous amino-yne click reaction has emerged as the method of choice for many researchers. By simply combining electron-deficient terminal alkynes with amines at room temperature without any catalyst, this reaction notably produces regio- and stereo-selective products (β-aminoacrylates) in excellent yields (Scheme 1) [20]. The mechanism underlying this reaction involves the non-bonding electron pair of nitrogen attacking the terminal carbon of an activated ethynyl group. This occurs due to the electron-withdrawing natural ability of the ester functional group, facilitating a nucleophilic addition step that leads to regioselectivity in the resulting product. From a thermodynamic point of view, *E*-isomers exhibit greater stability than *Z*-isomers during the proton transfer step. However, at room temperature, *Z*-isomers can be transformed to *E*-isomers through a single transition state involving “nitrogen activated double bond rotation”, thereby demonstrating the stereo-selectivity in the resulting enamine [21].

The amino-yne click reaction presents three additional advantages alongside the general benefits of click reactions: (I) It proceeds spontaneously at room temperature, requiring neither catalyst nor stimuli, thus simplifying the operational process. (II) Amines are very economical and accessible reagents, and propiolate-like compounds are easily synthesized from commercially available propiolic acid and aromatic or aliphatic alcohols by using esterification procedures under mild reaction conditions [20,44]. Moreover, the amino-containing biomolecules do not require premodification steps, as amino functional groups are frequent in a wide range of compounds, facilitating the experimental process and paving the way for the widespread utilization of the amino-yne click reactions across various domains. (III) The β-aminoacrylate resulting from the amino-yne reaction can respond to particular stimuli, such as weak acid media, causing the cleavage of the product and reobtaining the amino group, which make this type of reaction an interesting approach for designing materials that exhibit stimulus-triggered responses [21].

Bioconjugation can be considered as an intersection between chemistry and molecular biology, encompassing the covalent linking of synthetic or natural labels to biomolecular structures [45]. This interdisciplinary field focuses on biocompatible reactions that selectively, rapidly, and efficiently join substrates with biomolecules. Indeed, challenges like attaching small molecular probes (such as fluorescent dyes), radical probes, or affinity tags to biopolymers or linking complex carbohydrates with peptides can be possible thanks to click reactions, notably amino-yne click reactions. However, the alteration in biomolecules can affect their specific biological functions, so maintaining their inherent bioactivity once attached to the non-biological material poses a significant challenge [30]. In addressing this challenge, the bioconjugation of antibiotics onto various surfaces has garnered significant attention, aiming to design antimicrobial surfaces that mitigate infection-related complications associated with biomaterial implantation. Among these antibiotics, amoxicillin falls within the category of β-lactam amino-penicillin drugs, known for their wide action against both aerobic and anaerobic bacteria, exhibiting excellent absorption and tissue penetration capabilities [46]. Their mechanism of action involves disrupting the proper formation of the bacteria cell wall, leading to the death of susceptible microorganisms [47]. To achieve this, amoxicillin acts by inhibiting one or more enzymes, often referred to as penicillin-binding proteins (PBPs), which play a crucial role in the biosynthetic pathway of bacterial peptidoglycan [48]. As a result of this inhibition, the bacterial cell wall weakens, commonly followed by cell lysis and subsequent death [49,50]. Moreover, the structure–activity relationship (SAR) of these drug indicated that the main pharmacophore is 6-APA (6-aminopenicillanic acid) along with amide and aromatic ring moieties. Importantly, different works have reported that any substitution in the heteroaromatic ring tends to significantly diminish the activity of the drug, particularly when it comes to combating Gram-negative organisms like *Escherichia coli*. Furthermore, it has to be noted that these main functional groups, which confer antibacterial activity, are not modified by amino-yne click reactions, as only the amino takes a pivotal role in the reaction, and its modification does not imply a decrease in drug activity [46,47,49,50].

On the other hand, antimicrobial peptides (AMPs) represent a diverse class of small peptides present in nature, constituting an integral component of the innate immune defense across various organisms. AMPs exhibit a remarkable array of inhibitory actions, showcasing potent effects against a spectrum of pathogens including bacteria, fungi, parasites, and viruses. This broad-spectrum activity significantly enhances the appeal of these peptides as possible candidates in addressing microbial threats [51]. In this way, Feng et al. [52] investigated the impact of tryptophan within truncated dCATHs peptides derived from animal sources and classified as AMPs. Their findings underscored the pivotal role of tryptophan (TRP), highlighting that peptides incorporating this amino acid exhibited notably heightened antibacterial efficacy. Conversely, peptides devoid of tryptophan showcased a lack of discernible antibacterial activity. TRP, an indispensable amino acid sourced from plants, is pivotal in the in vivo synthesis of proteins. Among the 20 naturally occurring amino acids crucial for animal and human nutrition, TRP stands out for its multifaceted roles [53]. Functioning as an essential amino acid vital for normal growth, TRP assumes a dynamic role as a precursor in the synthesis of various bioactive compounds. Notably, it serves as a starting compound for an array of important molecules, including nicotinamide (vitamin B6), serotonin, melatonin, tryptamine, kynurenine, 3-hydroxykynurenine, and quinolinic and xanthurenic acids [54]. As the most widely dispersed indole derivative in nature, tryptophan holds the distinction of being the first recognized essential amino acid for numerous animal species. The implications of tryptophan and its metabolites span across a broad spectrum of health-related fields [55].

In this article, we propose a novel approach utilizing an amino-containing antibiotic for bioconjugation onto PLLA through an amino-yne click reaction. This method involves the use of an activated acetylene with an electron-attracting group attached to the carbon of the alkyne group while specifically employing a propiolate derivative that is a commercially available and easily modifiable reagent. Our study aims to establish a technique for immobilizing biologically active molecules onto polymer surfaces by employing an alkyne-activated amino click reaction. The surface used, PLLA, was fabricated and subjected to various chemical functionalizations to enable the covalent attachment of the propiolic derivative. In order to validate the proposed methodology and study its scope, before immobilizing amoxicillin, these alkyne-activated polymeric surfaces were reacted with an amino acid fluorophore compound (tryptophan), offering a straightforward approach to immobilizing diverse molecules through a simple modification.

## 2. Materials and Methods

### 2.1. Materials

Poly-L-lactide (PLLA) (Corbion, Amsterdam, The Netherlands) was used to prepare films with a thickness of 0.7 µm. Chloroform (>98%, Macron Fine Chemicals, Gliwice, Poland), methanol (MeOH, 98%, Panreac, Damrstadt, Germany), acetic acid (>98%, Sigma Aldrich, Steinheim am Albuch, Germany), hydrochloric acid (HCl, 37%, Panreac, Darmstadt, Germany), and Milli-Q water were used as solvents. Sodium hydroxide (99%, Panreac, Darmstadt, Germany), sodium hydrogen carbonate (NaHCO_3_, 99%, Merck, Darmstadt, Germany), sodium carbonate (Na_2_CO_3_, 98%, Panreac, Darmstadt, Germany), *N*-(3-dimethylaminopropyl)-*N-*ethylcarbodiimide hydrochloride (EDC·HCl, 98%, Sigma Aldrich, Beijing, China), *N*-hydroxysuccinimide (NHS, 98%, Sigma Aldrich, St. Louis, MO, USA), propiolic acid (95%, Sigma Aldrich, Poland), (S)-(-)-tryptophan (98%, Panreac, Darmstadt, Germany), and amoxicillin (98%, Sigma Aldrich, St. Louis, MO, USA) were used as reagents for the PLLA surface preactivation and functionalization.

### 2.2. Surface Preactivation and Drug Immobilization

Prior to drug immobilizations, PLLA films were fabricated and hydrolyzed with NaOH (1M) for 30 min at 50 °C. Afterwards, the samples were rinsed with HCl (10% *v*/*v*) and cleaned with water, obtaining hydrolyzed PLLA surfaces (PLLA-COOH). Following this, an amidation reaction with ethylendiamine was carried out. For this purpose, preactivated PLLA-COOH samples were introduced in a previously prepared EDC·HCl/NHS solution as described in our previous work [56]. Once the PLLA-NH_2_ surfaces were obtained, they were submitted to a further amidation reaction with previously activated propiolic acid. For that, propiolic acid (85 µL, 1.35 mmol) was treated with EDC·HCl (0.25 g, 1.40 mmol) and NHS (0.15 g, 1.40 mmol) in a phosphate buffer solution (pH 5) at room temperature for 4 h. After that, PLLA-NH_2_ samples were introduced into the propiolic-activated solution and were stirred at room temperature for 24 h. After that, PLLA-Alkyne samples were rinsed with water and further prepared for drug immobilization. Finally, PLLA-Alkyne surfaces were functionalized with either tryptophan or amoxicillin (1.4 mmol) previously dissolved in a Na_2_CO_3_ (0.1 M, 10 mL) solution at room temperature for 72 h. 

### 2.3. X-ray Photoelectron Spectroscopy (XPS)

The elemental analysis of pristine PLLA and amidated PLLA surfaces was performed by using an XPS SPECS system (SPECS Surface Nano Analysis, Berlin, Germany) with a focused monochromatic radiation source (500) with a dual anode (Al/Ag). It has to be noted that the equipment was furnished with a 150 1D-DLD analyzer (Phoibos, Berlin, Germany). PLLA samples were fixed with stainless steel holders and carbon tape during the measurements. 

### 2.4. Attenuated Total Reflection Fourier-Transform Infrared (ATR-FTIR) Spectroscopy

Surface chemical modifications of pristine PLLA and functionalized PLLA were analyzed via FTIR by employing an NICOLET Nexus 670 FT-IR spectrophotometer (Thermo Scientific, Loughborough, UK) equipped with an ATR employing 32 scans from 4000 cm^−1^ to 660 cm^−1^ within the wavenumber range and a resolution of 4 cm^−1^.

### 2.5. Scanning Electron Microscopy (SEM)

Morphological observations were carried out on functionalized PLLA surfaces by employing a HITACHI S-4800 (Singapore, Japan) electronic microscope. Different voltages were applied on the PLLA surfaces: an electron acceleration voltage of 1.5 kV over a distance of 7.1 mm × 5.00 k for the pristine PLLA and PLLA-COOH surfaces, an electron acceleration voltage of 1.5 kV over a distance of 8.3 mm × 5.00 k for the PLLA-NH2 surface, 2.0 kV over a distance of 8.3 mm × 5.00 k for the PLLA-Alkyne, 2.0 kV over a distance of 7.2 mm × 5.00 k for the PLLA-TRP, and 1.5 kV over a distance of 6.0 mm × 5.00 k for the PLLA-Amox surface. 

### 2.6. Water Contact Angle (WCA)

The wettability of different PLLA films, including pristine PLLA, hydrolyzed PLLA, amidated PLLA, tryptophan-immobilized PLLA and amoxicillin-immobilized PLLA, were analyzed using the static contact angle method (NEURTEK Instruments OCA 15 EC, Eibar, Spain). Milli-Q water was used as a testing liquid, and the sessile drop method (3 µL per drop) was carried out at room temperature to conduct the measurements. The average values were calculated using eight measurements of each composition. 

### 2.7. Confocal Fluorescence

The fluorescence emissions of the pristine PLLA and the tryptophan-immobilized PLLA surfaces were analyzed using a Zeiss Axioskop epifluorescence microscope (Jena, Germany).

### 2.8. Fluorescence Spectroscopy

Emission spectra of the PLLA films were recorded exciting in the UV region using an Edinburgh Instruments spectrofluorimeter (FLSP920 model, Livingston, UK) in a front-face configuration. Samples were positioned at 40° and 50° angles to the excitation and emission beams, respectively, and were tilted at a 30° angle relative to the plane formed by the direction of incidence and detection.

## 3. Results

### 3.1. PLLA Surface Preactivation and Tryptophan Immobilization

**Scheme 2:**
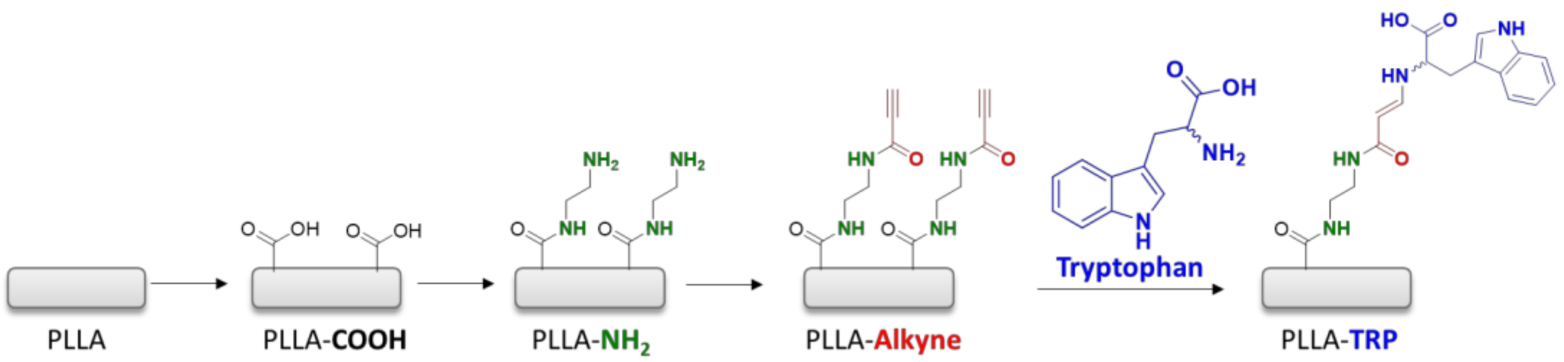
Chemical PLLA surface-modification steps for tryptophan immobilization.

Before performing the immobilization of the amino-containing molecules (that is, tryptophan and amoxicillin), it was necessary to accomplish some modifications to the PLLA surfaces. Initially, basic hydrolysis and subsequent acidification of the PLLA surface induced the formation of carboxylic acids on the surface of the substrates (PLLA-COOH). Indeed, pristine PLLA was submerged into a NaOH 1M solution for 30 min at 50 °C. Basic residual traces were rinsed with water and next with HCl solution. Following hydrolysis, two sequential amidation steps were carried out. The first involved the functionalization with ethylendiamine (ETDA), resulting in the terminal amino functional group (PLLA-NH_2_). In the subsequent amidation, the necessary alkyne group for the click reaction was incorporated (PLLA-Alkyne). For this purpose, propiolic acid was employed as an electron-withdrawing alkyne. Ultimately, the fluorescent tryptophan was immobilized via an amino-yne click reaction (PLLA-TRP) (Scheme 2). 

**Figure 1:**
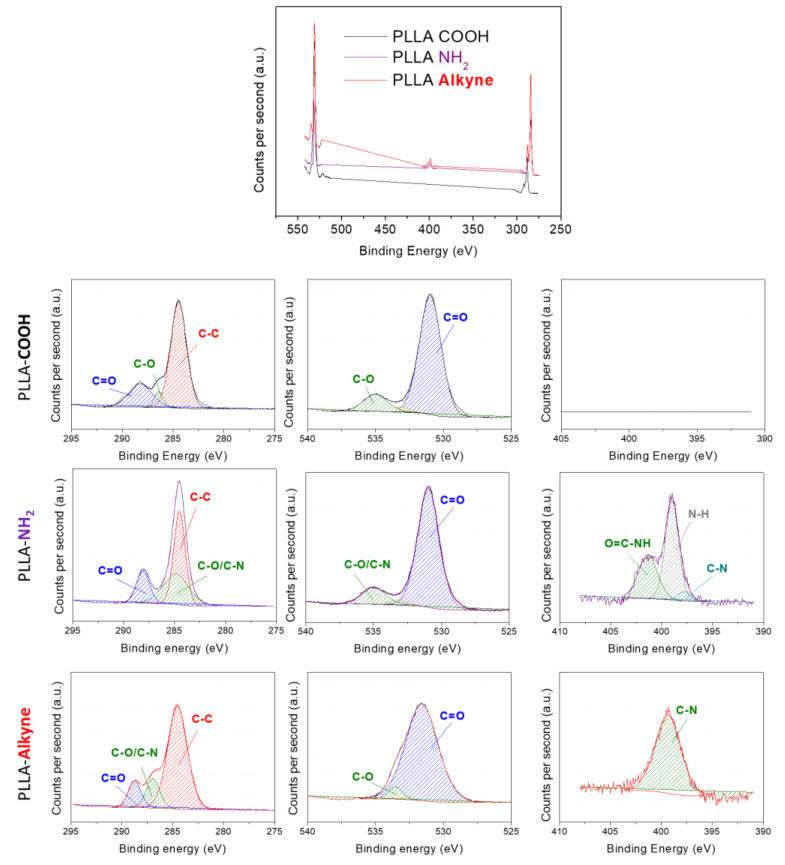
XPS total spectra of PLLA-COOH, PLA-NH_2_, and PLLA-Alkyne and the deconvolution spectra of C1s, O1s, and N1s of each sample.

The chemical composition of each functionalization step was analyzed by using XPS. As can be seen in Figure 1, PLLA-COOH exhibited two major peaks at 284.6 eV and 535 eV, corresponding to C1s (69.9%) and O1s (29.8%), respectively. Meanwhile, as was expected, the high-resolution spectra of carbon showed three peaks corresponding to C-C/C-H (284.6 eV), C-O (286.3 eV), and C=O (288.8 eV) functional groups found in the chemical structure of PLLA, which agreed with the literature [57,58,59]. After amidation with ethylendiamine, a new peak at 400 eV attributed to N1s appeared in the spectrum of PLLA-NH_2_, confirming the successful amidation of the hydrolyzed PLLA. Additionally, high-resolution carbon spectra also corroborated the introduction of nitrogen due to the enhanced peak of the C-O/C-N contribution at 285.8 eV. The contents of C, O, and N elements in PLLA-NH_2_ were 63.1%, 31.5% and 1.8%, respectively. Following the second amidation process employing propiolic acid (PLLA-Alkyne), similar C, O, and N content values were obtained when compared to PLLA-NH_2_ (62.0%, 26.0%, and 3.2%, respectively). However, notable changes were detected in the nitrogen high-resolution spectra of PLLA-NH_2_ and PLLA-Alkyne. Indeed, the contributions observed at 398.9 eV, 399.7 eV, and 400.6 eV corresponding to the emergence of NH_2_, N-C=O, and C-NH, respectively, on the PLLA-NH_2_ surfaces disappeared, while the NH-C=O contribution increased on the PLLA-Alkyne surfaces. These findings confirmed the introduction of propiolic acid, a terminal alkyne ester crucial for facilitating catalyst-free amino-yne click reactions.

**Figure 2:**
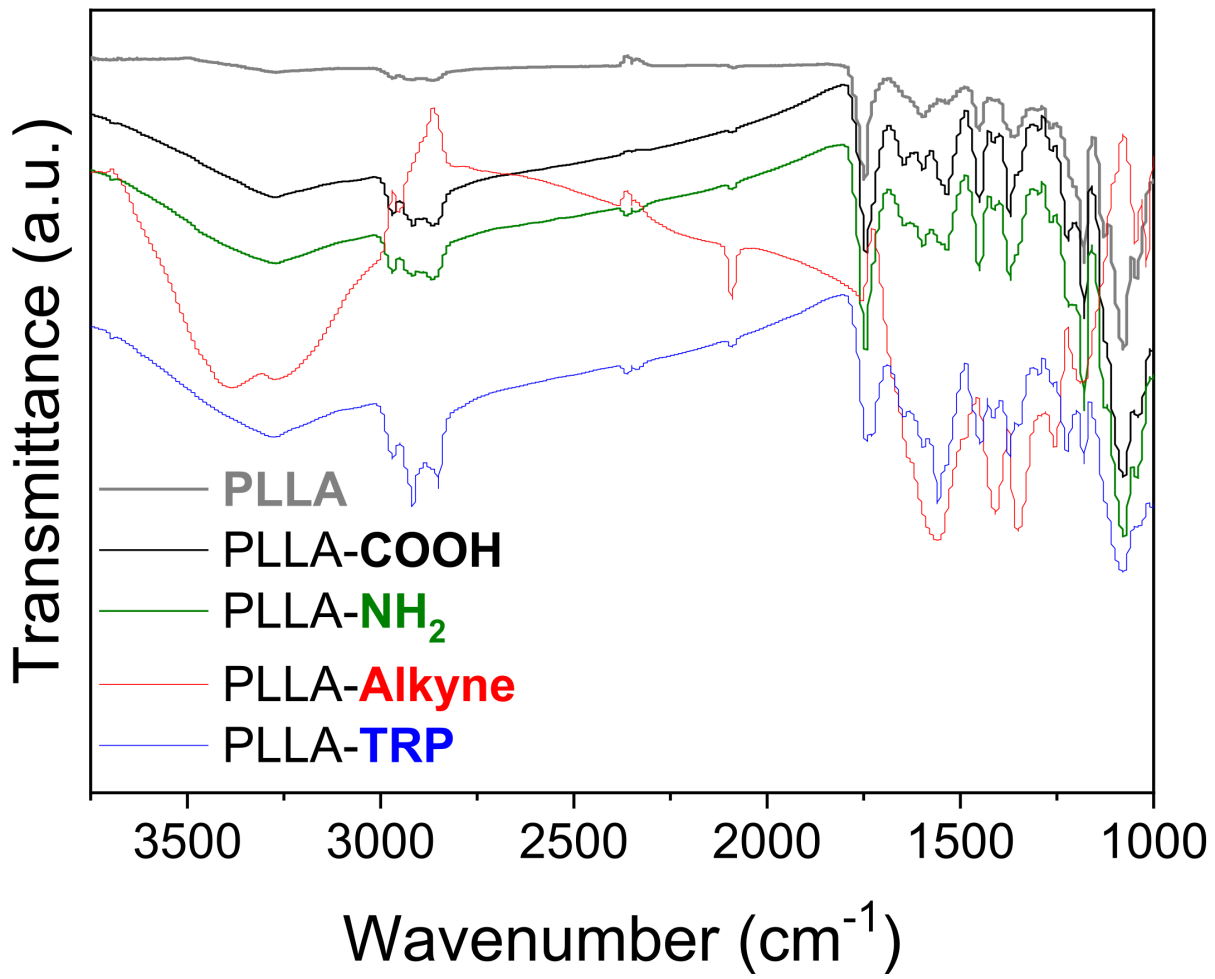
ATR-FTIR spectra of pristine PLLA and functionalized PLLA, including PLLA-TRP.

After every functionalization step, the surface chemical structure was determined by means of an ATR-FTIR technique (Figure 2). ATR-FTIR measurements of the pristine PLLA sample revealed mainly a prominent signal at 1700 cm^−1^ corresponding to the stretching vibration of the C=O bond, thus demonstrating the presence of ester groups within the polymer structure. Additionally, the observed signals between 2850–2900 cm^−1^, which were attributed to the C-H stretching band, further confirmed the presence of CH_3_ and CH_2_ groups in the polymer structure. Upon hydrolysis, a new signal emerged around 3300 cm^−1^, indicative of the vibrational stretch of the O-H bond and corresponding to the formation of new functional groups on the surface (specifically, carboxylic acids). Following the amidation process with ethylendiamine, no significant changes were observed, as the N-H and O-H bonds displayed similarities in their bond stretching. Nevertheless, subsequent amidation with propiolic acid revealed two new signals at 3300 cm^−1^ and 2100 cm^−1^ corresponding to the terminal C_sp_H and C_sp_-C_sp_ bonds, respectively. These results indicated the introduction of terminal alkyne groups onto the PLLA surfaces. After the conjugation reaction with tryptophan (PLLA-TRP), this last particular signal disappeared, corroborating that the amino-yne click reaction between the tryptophan and functionalized PLLA had been successfully carried out. Moreover, due to the presence of tryptophan at the top of the surface, the signals corresponding to O-H and N-H suffered a slight decrease. Thus, it confirmed again that the reaction took place successfully.

**Figure 3:**
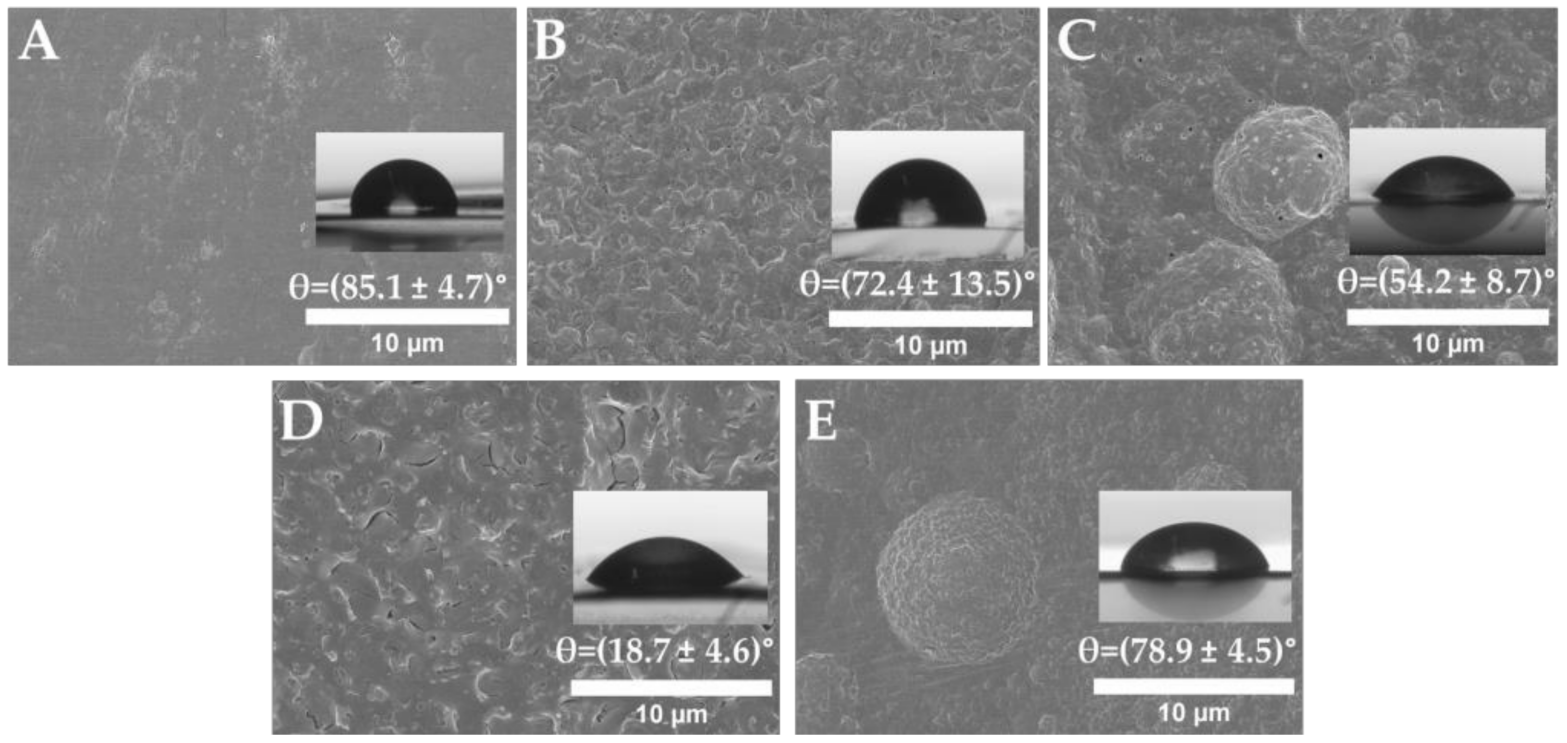
SEM images of functionalized PLLA: (**A**) pristine PLLA, (**B**) PLLA-COOH, (**C**) PLLA-NH_2_, (**D**) PLLA-Alkyne, and (**E**) PLLA-TRP analyzed at 10 µm.

Many studies, including our previous work, confirmed that surface chemical modification can alter the morphology and wettability properties of a surface [10,26,56]. Therefore, SEM and contact angle techniques were employed after every modification step in order to determine the topographical changes and water affinity of the surface (Figure 3).

According to the static water contact angle values (WCA), as was expected, the non-functionalized PLLA presented a slightly hydrophobic WCA value of around 85.1°. Since the pristine PLLA exhibited a lack of hydrophilic active functional groups on the surface, after the hydrolysis reaction, the WCA value decreased to 72.4°, resulting in a more hydrophilic surface. In fact, these results indicated that the formation of polar groups on the surface of the PLLA had been achieved, as both the carboxylic acid and hydroxyl group could generate hydrogen bonds with the deposited water droplets, making the droplet less spherical and covering more surface area, thereby decreasing the WCA value. After amidation reaction with ethylendiamine, the WCA decreased again considerably to 52.3°, suggesting that the surface exhibited more affinity to water, as functional groups capable of forming hydrogen bonds appeared at the end of the surface (N-H bonds in particular). Once amidated with propiolic acid, the WCA value kept decreasing until reaching 18.7°. Although the introduction of the less polar group (alkyne) could have diminished the hydrogen bond interactions between the droplet and the material, the roughness of the material could explain the low value of the WCA. Indeed, according to the Wenzel state, the roughness of the surface amplified the intrinsic wetting properties [60]. Finally, after tryptophan immobilization, the WCA value increased to 78.9°; this increment in the WCA value corroborated the presence of aromatic groups on the polymer surface. Therefore, this enhanced wettability change in the WCA value corroborated that tryptophan conjugation was successfully carried out. 

On the other hand, SEM images indicated the surface morphologies of different PLLA samples, as can be seen in Figure 3. Regarding the topographical properties, the pristine PLLA surfaces were quite flat and smooth, but some breaks appeared due to the pressing process suffered in film fabrication. After hydrolysis, the surface became rougher, and slight changes were exhibited after amidation with ethylendiamine. Once amidated with propiolic acid, new protrusions appeared on the surface. In fact, as previously mentioned, this change in the surface topography was probably responsible for the increase in the hydrophilicity of this surface. Finally, upon the amino acid immobilization, the surface became smoother, although the presence of some cavities was observed. These particular surface variations indicated again that surface functionalization and tryptophan immobilization were actually achieved. 

**Figure 4:**
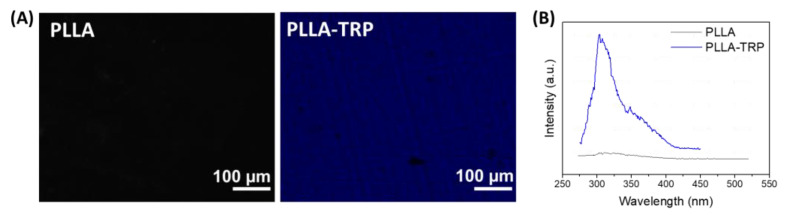
(**A**) Fluorescence microscopy images of pristine PLLA and tryptophan-functionalized PLLA; (**B**) fluorescence spectroscopy spectra of PLLA and PLLA-TRP.

Finally, by means of confocal fluorescence and fluorescence spectroscopy, the conjugation of tryptophan via an amino-yne click reaction was corroborated (Figure 4). Both experiments demonstrated the successful immobilization of tryptophan when the sample was irradiated under different wavelengths. This fluorophore presented an excitation peak at 225–280 nm and an emission around 320–370 nm. When both the pristine PLLA sample and the PLLA-TRP sample were excited under the same energy value, only the PLLA-TRP emitted a significant blue light corresponding to the emission fluorescence range of tryptophan, indicating that the amino acid had been successfully immobilized. Moreover, fluorescence spectroscopy corroborated the previous fluorescence emission. In this experiment, both samples (pristine PLLA and PLLA-TRP) were excited at the same wavelength, but, as expected, only the PLLA-TRP showed an emission peak around 310 nm resulting from the tryptophan immobilization on surface. 

### 3.2. Amoxicillin Immobilization onto the PLLA Surfaces via a Copper-Free Amino-Yne Click Reaction

**Scheme 3:**
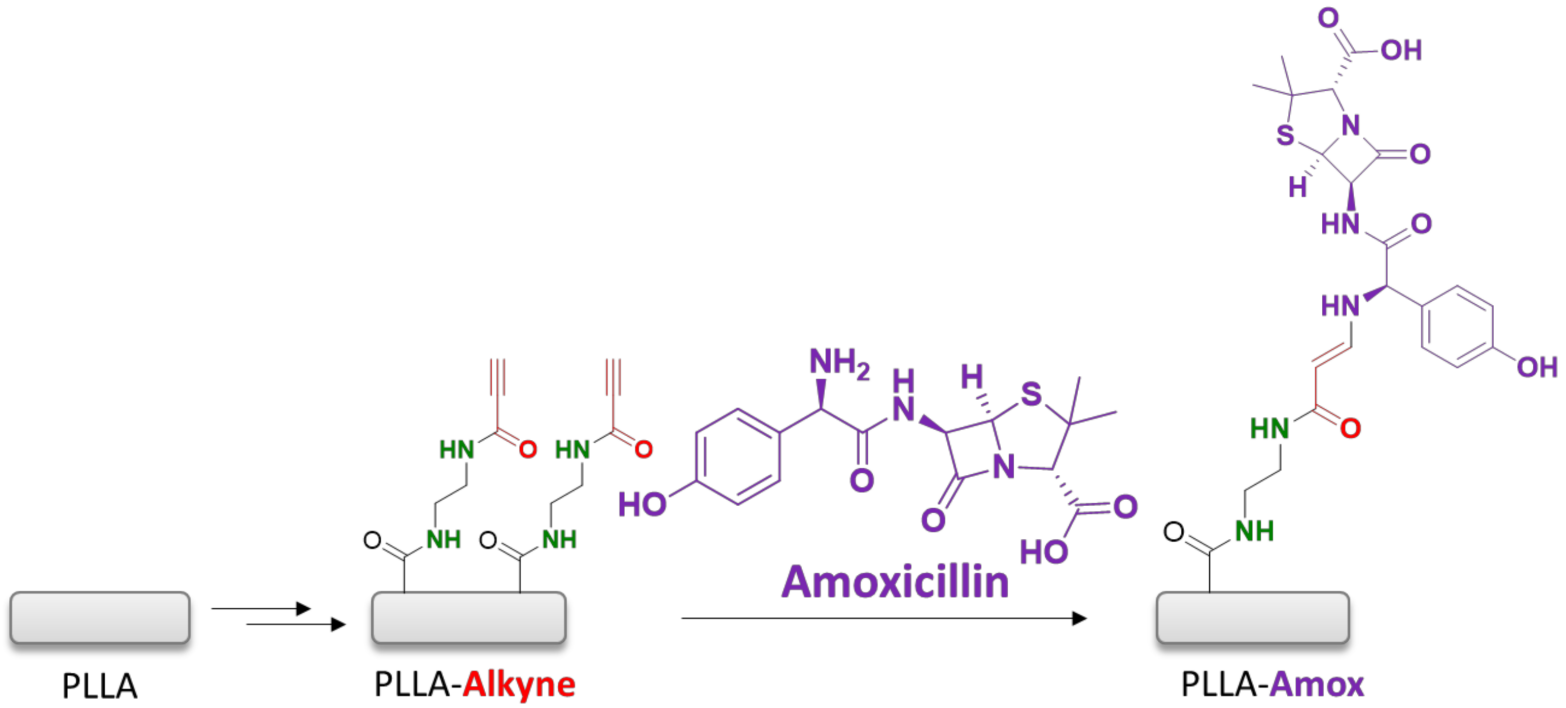
Amoxicillin immobilization via an amino-yne click reaction onto the PLLA surface.

The excellent results obtained in the immobilization of the fluorophore encouraged us to use the same methodology to bioconjugate the antibiotic amoxicillin on the polymeric surface (Scheme 3).

**Figure 5:**
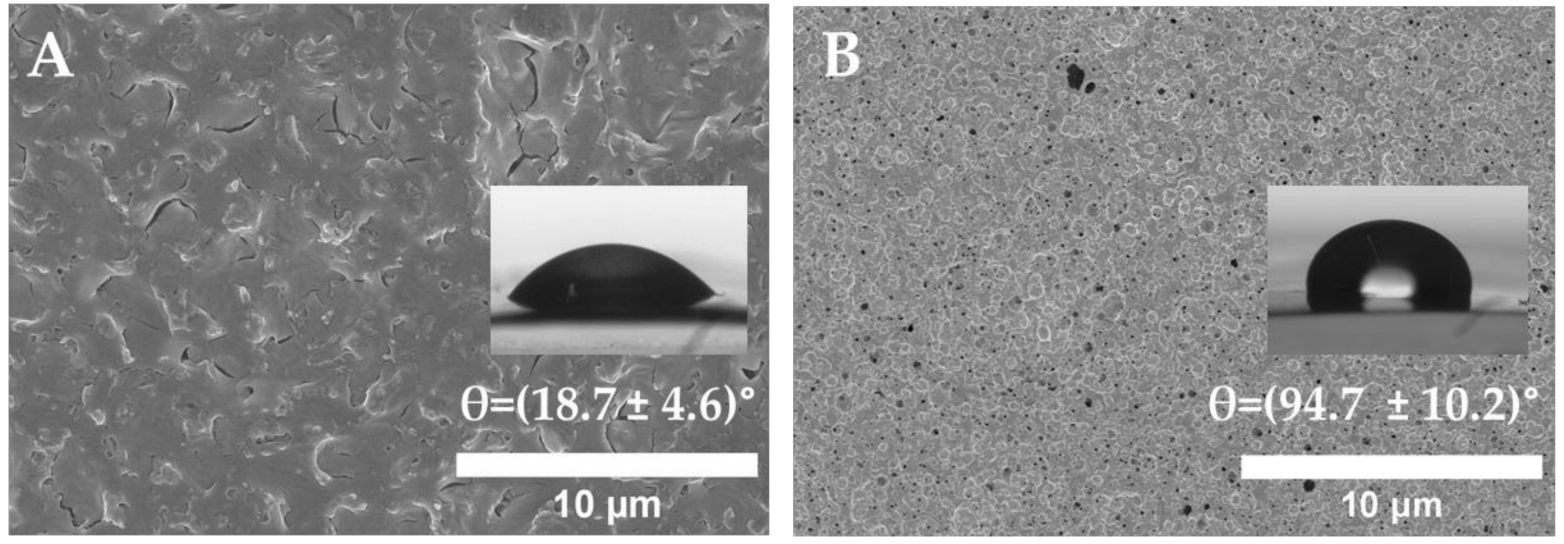
SEM images and WCA value of (**A**) PLLA-Alkyne and (**B**) PLLA-Amox.

For the tryptophan immobilization, amoxicillin bioconjugation required previous surface functionalization including hydrolysis, amidation, and propiolic acid chemical introduction. When incorporating amoxicillin onto the surface, the wettability and topography were also studied (Figure 5). 

Indeed, when comparing the WCA value of the propiolated (PLLA-Alkyne) and amoxicillin bioconjugates (PLLA-Amox), differences could be observed. The WCA value significantly increased to 94.7°, making the surface more hydrophobic than the tryptophan-immobilized surface. In fact, amoxicillin presented an aminopenicillanic acid ring (APA), two fused rings, and a phenyl functional group. These structural elements rendered the surface less susceptible to forming hydrogen bonds. Therefore, the hydrophobic interactions on amoxicillin were higher compared to tryptophan, which only posed the indole-aromatic group. On the other hand, granular roughness could be observed on the amoxicillin-functionalized PLLA surfaces, but they were smoother than in the previous functionalization. These changes in wettability and topography indicated that amoxicillin immobilization was carried out.

**Figure 6:**
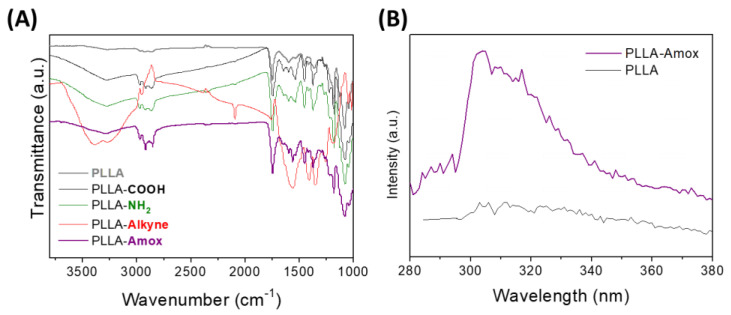
(**A**) ATR-FTIR analysis of pristine PLLA and functionalized PLLA, including PLLA-Amox; (**B**) fluorescence spectroscopy spectra of pristine PLLA and PLLA-Amox.

Further analyses were performed to corroborate the chemical bioconjugation of amoxicillin. Indeed, ATR-FTIR and fluorescence spectroscopy were employed. As commented previously, the introduction of the alkyne onto the PLLA surface was confirmed by the characteristic signals observed at 3300 cm^−1^ and 2100 cm^−1^. In fact, once amoxicillin was immobilized onto the modified PLLA surface via an amino-yne click reaction, the signal related to C_sp_-C_sp_ and C_sp_H bonds disappeared, confirming that the alkyne reacted with the amino moiety of the antibiotic (Figure 6). These findings indicated that bioconjugation of amoxicillin via the amino-yne click reaction was obtained.

Moreover, fluorescence assays performed by using spectroscopic methods confirmed again the immobilization of the antibiotic compound. In fact, the non-functionalized PLLA and PLLA-Amox samples were excited in the same wavelength range, but only the emission spectra of the PLLA-Amox exhibited a significant signal at 310 nm, indicating that amoxicillin had been successfully bioconjugated on the PLLA surfaces (Figure 6).

## 4. Conclusions

The development of a surface-treatment strategy for the conjugation of biological compounds onto a polymer surface using catalyst-free amino-yne click chemistry was achieved in this study. The success of the surface prefunctionalization and alkyne activation was confirmed through XPS and ATR-FTIR analyses. Indeed, the XPS results indicated that the hydrolysis and subsequent amidation processes were carried out, since high-resolution spectra of C and N exhibited the contributions of different species. On the other hand, ATR-FTIR corroborated the addition of alkyne through the presence of the peaks at 2100 cm^−1^ related to the C≡C bond and at 3300 cm^−1^ corresponding to C_sp_-H. Additionally, ATR-FTIR spectroscopy, water contact angles, SEM, and fluorescence analysis confirmed the immobilization of both amino-moiety-containing molecules: tryptophan (employed as pharmacophore in order to validate the methodology) and the antibiotic amoxicillin. 

Hence, this work provides an efficient route to conjugate biological compounds without requiring a metal catalyst, which overcomes the drawbacks of the use of copper, such as toxicity. Furthermore, derivatization of the compound to be immobilized is not necessary as long as it has a nucleophilic amino group. This versatile approach to immobilization exhibits great potential in different areas, such as materials science, organic chemistry, polymer science, and especially in the biomedical field. The applications of this approach are extensive. For instance, it enables the creation of biofunctional materials suitable for a diverse array of biomedical applications ranging from implant coatings to the development of biosensors and advancements in tissue engineering. Moreover, any drug or biologically active polypeptide featuring a free amino functional group can be immobilized onto a wide range of surfaces without requiring previous compound derivatization and in the absence of any catalyst. Therefore, this advancement significantly expands the scope of bioconjugation possibilities for compounds possessing a free amino functional group. 

## Data Availability

Data are contained within the article.

## References

[1] Taib N.A.A.B., Rahman M.R., Huda D., Kuok K.K., Hamdan S., Bakri M.K.B., Julaihi M.R.M.B., Khan A. (2022). A Review on Poly Lactic Acid (PLA) as a Biodegradable Polymer.

[2] Garlotta D. (2019). A Literature Review of Poly(Lactic Acid). J. Polym. Environ..

[3] Mehta R., Kumar V., Bhunia H., Upadhyay S.N. (2005). Synthesis of poly(lactic acid): A review. J. Macromol. Sci. Polym. Rev..

[4] Scaffaro R., Lopresti F., Marino A., Nostro A. (2018). Antimicrobial additives for poly(lactic acid) materials and their applications: Current state and perspectives. Appl. Microbiol. Biotechnol..

[5] Seyednejad H., Ghassemi A.H., Van Nostrum C.F., Vermonden T., Hennink W.E. (2011). Functional aliphatic polyesters for biomedical and pharmaceutical applications. J. Control. Release.

[6] Chen H.L., Chung J.W.Y., Yan V.C.M., Wong T.K.S. (2023). Polylactic Acid-Based Biomaterials in Wound Healing: A Systematic Review. Adv. Ski. Wound Care.

[7] Narayanan G., Vernekar V.N., Kuyinu E.L., Laurencin C.T. (2016). Poly (Lactic Acid)-Based Biomaterials for Orthopaedic Regenerative Engineering. Adv. Drug Deliv. Rev..

[8] Lasprilla A.J.R., Martinez G.A.R., Lunelli B.H., Jardini A.L., Filho R.M. (2012). Poly-lactic acid synthesis for application in biomedical devices—A review. Biotechnol. Adv..

[9] Mu M., Liu S., DeFlorio W., Hao L., Wang X., Salazar K.S., Taylor M., Castillo A., Cisneros-Zevallos L., Oh J.K. (2023). Influence of Surface Roughness, Nanostructure, and Wetting on Bacterial Adhesion. Langmuir.

[10] Sánchez-Bodón J., Diaz-Galbarriatu M., Pérez-Álvarez L., Moreno-Benítez I., Vilas-Vilela J.L. (2023). Strategies to Enhance Biomedical Device Performance and Safety: A Comprehensive Review. Coatings.

[11] Olmo J.A.D., Ruiz-Rubio L., Pérez-Alvarez L., Sáez-Martínez V., Vilas-Vilela J.L. (2020). Antibacterial coatings for improving the performance of biomaterials. Coatings.

[12] Pérez Davila S., González Rodríguez L., Chiussi S., Serra J., González P. (2021). How to sterilize polylactic acid based medical devices?. Polymers.

[13] Subbiahdoss G., Kuijer R., Grijpma D.W., van der Mei H.C., Busscher H.J. (2009). Microbial biofilm growth vs. tissue integration: “The race for the surface” experimentally studied. Acta Biomater..

[14] Bhagwat G., O’Connor W., Grainge I., Palanisami T. (2021). Understanding the Fundamental Basis for Biofilm Formation on Plastic Surfaces: Role of Conditioning Films. Front. Microbiol..

[15] Arciola C.R., Campoccia D., Montanaro L. (2018). Implant infections: Adhesion, biofilm formation and immune evasion. Nat. Rev. Microbiol..

[16] Rana D., Ramasamy K., Leena M., Pasricha R., Manivasagam G., Ramalingam M. (2017). Surface Functionalization of Biomaterials.

[17] Pujari S.P., Scheres L., Marcelis A.T.M., Zuilhof H. (2014). Covalent surface modification of oxide surfaces. Angew. Chem. Int. Ed..

[18] Zhang L.C., Chen L.Y., Wang L. (2020). Surface Modification of Titanium and Titanium Alloys: Technologies, Developments, and Future Interests. Adv. Eng. Mater..

[19] Xu J., Zhang J., Shi Y., Tang J., Huang D., Yan M., Dargusch M.S. (2022). Surface Modification of Biomedical Ti and Ti Alloys: A Review on Current Advances. Materials.

[20] He B., Su H., Bai T., Wu Y., Li S., Gao M., Hu R., Zhao Z., Qin A., Ling J. (2017). Spontaneous Amino-yne Click Polymerization: A Powerful Tool toward Regio- and Stereospecific Poly(β-aminoacrylate)s. J. Am. Chem. Soc..

[21] Zhang J., Zhang Z., Wang J., Zang Q., Sun J.Z., Tang B.Z. (2021). Recent progress in the applications of amino-yne click chemistry. Polym. Chem..

[22] Li H.-C., Sun X.-M., Huang Y.-R., Peng Y.-H., Liu J., Ren L. (2022). Synthetic Crosslinker Based on Amino–yne Click to Enhance the Suture Tension of Collagen-Based Corneal Repair Materials. ACS Appl. Polym. Mater..

[23] Poonia M., Küster T., Bothun G.D. (2022). Organic Anion Detection with Functionalized SERS Substrates via Coupled Electrokinetic Preconcentration, Analyte Capture, and Charge Transfer. ACS Appl. Mater. Interfaces.

[24] Li Q., Yin G., Wang J., Li L., Liang Q., Zhao X., Chen Y., Zheng X., Zhao X. (2022). An emerging paradigm to develop analytical methods based on immobilized transmembrane proteins and its applications in drug discovery. TrAC Trends Anal. Chem..

[25] Chen C., Zhang S.M., Lee I.S. (2013). Immobilizing bioactive molecules onto titanium implants to improve osseointegration. Surf. Coatings Technol..

[26] Sánchez-Bodón J., Andrade-Del Olmo J., Alonso J.M., Moreno-Benítez I., Vilas-Vilela J.L., Pérez-Alvarez L. (2022). Bioactive Coatings on Titanium: A Review on Hydroxylation, Self-Assembled Monolayers (SAMs) and Surface Modification Strategies. Polymers.

[27] Zhang Y., Shen J., Hu R., Shi X., Hu X., He B., Qin A., Tang B.Z. (2020). Fast surface immobilization of native proteins through catalyst-free amino-yne click bioconjugation. Chem. Sci..

[28] Kolb H.C., Finn M.G., Sharpless K.B. (2001). Click Chemistry: Diverse Chemical Function from a Few Good Reactions. Angew. Chem. Int. Ed..

[29] Yoon H.Y., Lee D., Lim D.K., Koo H., Kim K. (2022). Copper-Free Click Chemistry: Applications in Drug Delivery, Cell Tracking, and Tissue Engineering. Adv. Mater..

[30] Amna B., Ozturk T. (2021). Click chemistry: A fascinating method of connecting organic groups. Org. Commun..

[31] Chen Y., Tong Z.-R., Chen Y., Tonfg Z.-R. (2017). Click Chemistry: Approaches, Applications, and Challenges.

[32] Rostovtsev V.V., Green L.G., Fokin V.V., Sharpless K.B. (2002). A Stepwise Huisgen Cycloaddition Process: Copper(I)-Catalyzed Regioselective “Ligation” of Azides and Terminal Alkynes. Angew. Chem. Int. Ed..

[33] Lutz J. (2007). 1,3-Dipolar Cycloadditions of Azides and Alkynes: A Universal Ligation Tool in Polymer and Materials Science. Angew. Chem. Int. Ed..

[34] Suárez A. (2012). Reacciones de cicloadición 1,3-dipolares a alquinos catalizadas por cobre. An. Quím.

[35] Binder W., Kluger C. (2006). Azide/Alkyne-“Click” Reactions: Applications in Material Science and Organic Synthesis. Curr. Org. Chem..

[36] Arslan M., Acik G., Tasdelen M.A. (2019). The emerging applications of click chemistry reactions in the modification of industrial polymers. Polym. Chem..

[37] Agrahari A.K., Bose P., Jaiswal M.K., Rajkhowa S., Singh A.S., Hotha S., Mishra N., Tiwari V.K. (2021). Cu(I)-Catalyzed Click Chemistry in Glycoscience and Their Diverse Applications. Chem. Rev..

[38] Wang C., Ikhlef D., Kahlal S., Saillard J.Y., Astruc D. (2016). Metal-catalyzed azide-alkyne “click” reactions: Mechanistic overview and recent trends. Coord. Chem. Rev..

[39] O’Hern C.I.Z., Djoko K.Y. (2022). Copper Cytotoxicity: Cellular Casualties of Noncognate Coordination Chemistry. mBio.

[40] Baskin J.M., Bertozzi C.R. (2007). Bioorthogonal click chemistry: Covalent labeling in living systems. QSAR Comb. Sci..

[41] Agard N.J., Prescher J.A., Bertozzi C.R. (2004). A strain-promoted [3 + 2] azide-alkyne cycloaddition for covalent modification of biomolecules in living systems. J. Am. Chem. Soc..

[42] Bach R.D. (2009). Ring strain energy in the cyclooctyl system. The effect of strain energy on [3 + 2] cycloaddition reactions with azides. J. Am. Chem. Soc..

[43] Sakata Y., Nabekura R., Hazama Y., Hanya M., Nishiyama T., Kii I., Hosoya T. (2022). Synthesis of Functionalized Dibenzoazacyclooctynes by a Decomplexation Method for Dibenzo-Fused Cyclooctyne-Cobalt Complexes. Org. Lett..

[44] Worch J.C., Stubbs C.J., Price M.J., Dove A.P. (2021). Click Nucleophilic Conjugate Additions to Activated Alkynes: Exploring Thiol-yne, Amino-yne, and Hydroxyl-yne Reactions from (Bio)Organic to Polymer Chemistry. Chem. Rev..

[45] Stump B. (2022). Click Bioconjugation: Modifying Proteins Using Click-Like Chemistry. ChemBioChem.

[46] Bodey G.P., Nance J. (1972). Amoxicillin: In Vitro and Pharmacological Studies. Antimicrob. Agents Chemother..

[47] De Marco B.A., Natori J.S.H., Fanelli S., Tótoli E.G., Salgado H.R.N. (2017). Characteristics, Properties and Analytical Methods of Amoxicillin: A Review with Green Approach. Crit. Rev. Anal. Chem..

[48] Salvo F., De Sarro A., Caputi A.P., Polimeni G. (2009). Amoxicillin and amoxicillin plus clavulanate: A safety review. Expert Opin. Drug Saf..

[49] Neu H.C. (1974). Antimicrobial Activity and Human Pharmacology of Amoxicillin. J. Infect. Dis..

[50] Goldstein E.C., Citron D.M. (1986). Comparative In Vitro Activities of Amoxicillin-Clavulanic Acid and Imipenem against Anaerobic Bacteria Isolated from Community Hospitals. Antimicrob. Agents Chemother..

[51] Huan Y., Kong Q., Mou H., Yi H. (2020). Antimicrobial Peptides: Classification, Design, Application and Research Progress in Multiple Fields. Front. Microbiol..

[52] Feng X., Jin S., Wang M., Pang Q., Liu C., Liu R., Wang Y., Yang H., Liu F., Liu Y. (2020). The Critical Role of Tryptophan in the Antimicrobial Activity and Cell Toxicity of the Duck Antimicrobial Peptide DCATH. Front. Microbiol..

[53] Palego L., Betti L., Rossi A., Giannaccini G. (2016). Tryptophan Biochemistry: Structural, Nutritional, Metabolic, and Medical Aspects in Humans. J. Amino Acids.

[54] Friedman M. (2018). Analysis, Nutrition, and Health Benefits of Tryptophan. Int. J. Tryptophan Res..

[55] Nayak B.N., Singh R.B., Buttar H.S. (2019). Role of tryptophan in health and disease: Systematic review of the anti-oxidant, anti-inflammation, and nutritional aspects of tryptophan and its metabolites. World Heart J..

[56] Sánchez-Bodón J., Ruiz-Rubio L., Hernáez-Laviña E., Vilas-Vilela J.L., Moreno-Benítez M.I. (2021). Poly(L-lactide)-based anti-inflammatory responsive surfaces for surgical implants. Polymers.

[57] Renò F., D’Angelo D., Gottardi G., Rizzi M., Aragno D., Piacenza G., Cartasegna F., Biasizzo M., Trotta F., Cannas M. (2012). Atmospheric pressure plasma surface modification of poly(D, L-lactic acid) increases fibroblast, osteoblast and keratinocyte adhesion and proliferation. Plasma Process. Polym..

[58] Sourkouni G., Kalogirou C., Moritz P., Gödde A., Pandis P.K., Höfft O., Vouyiouka S., Zorpas A.A., Argirusis C. (2021). Study on the influence of advanced treatment processes on the surface properties of polylactic acid for a bio-based circular economy for plastics. Ultrason. Sonochem..

[59] De Geyter N., Morent R., Desmet T., Trentesaux M., Gengembre L., Dubruel P., Leys C., Payen E. (2010). Plasma modification of polylactic acid in a medium pressure DBD. Surf. Coatings Technol..

[60] Ubuo E.E., Udoetok I.A., Tyowua A.T., Ekwere I.O., Al-Shehri H.S. (2021). The direct cause of amplified wettability: Roughness or surface chemistry?. J. Compos. Sci..

